# Association of Annual Intensive Care Unit Sepsis Caseload With Hospital Mortality From Sepsis in the United Kingdom, 2010-2016

**DOI:** 10.1001/jamanetworkopen.2021.15305

**Published:** 2021-06-29

**Authors:** Ritesh Maharaj, Alistair McGuire, Andrew Street

**Affiliations:** 1 Department of Health Policy, London School of Economics and Political Science, London, UK; 2 Department of Critical Care, Kings College Hospital NHS Foundation Trust, London, UK

## Abstract

**Importance:**

Sepsis is associated with a high burden of inpatient mortality. Treatment in intensive care units (ICUs) that have more experience treating patients with sepsis may be associated with lower mortality.

**Objective:**

To assess the association between the volume of patients with sepsis receiving care in an ICU and hospital mortality from sepsis in the UK.

**Design, Setting, and Participants:**

This retrospective cohort study used data from adult patients with sepsis from 231 UK ICUs between 2010 and 2016. Demographic and clinical data were extracted from the Intensive Care National Audit & Research Centre (ICNARC) Case Mix Programme database. Data were analyzed from January 1, 2010, to December 31, 2016.

**Exposures:**

Annual sepsis case volume in an ICU in the year of a patient’s admission.

**Main Outcomes and Measures:**

Hospital mortality after ICU admission for sepsis assessed using a mixed-effects logistic model in a 3-level hierarchical structure based on the number of individual patients nested in years nested within ICUs.

**Results:**

Among 273 001 patients included in the analysis, the median age was 66 years (interquartile range, 53-76 years), 148 149 (54.3%) were male, and 248 275 (91.0%) were White. The mean ICNARC-2018 illness severity score was 21.0 (95% CI, 20.9-21.0). Septic shock accounted for 19.3% of patient admissions, and 54.3% of patients required mechanical ventilation. The median annual sepsis volume per ICU was 242 cases (interquartile range, 177-334 cases). The study identified a significant association between the volume of sepsis cases in the ICU and mortality from sepsis; in the logistic regression model, hospital mortality was significantly lower among patients admitted to ICUs in the highest quartile of sepsis volume compared with the lowest quartile (odds ratio [OR], 0.89; 95% CI, 0.82-0.96; *P* = .002). With volume modeled as a restricted cubic spline, treatment in a larger ICU was associated with lower hospital mortality. A lower annual volume threshold of 215 patients above which hospital mortality decreased significantly was found; 38.8% of patients were treated in ICUs below this threshold volume. There was no significant interaction between ICU volume and severity of illness as described by the ICNARC-2018 score (β [SE], –0.00014 [0.00024]; *P* = .57).

**Conclusions and Relevance:**

The findings suggest that patients with sepsis in the UK have higher odds of survival if they are treated in an ICU with a larger sepsis case volume. The benefit of a high sepsis case volume was not associated with the severity of the sepsis episode.

## Key Points

**Question:**

Is there an association between the annual volume of sepsis cases in an intensive care unit (ICU) and hospital mortality from sepsis?

**Findings:**

In this cohort study of 273 001 patients with sepsis at 231 ICUs in the UK, a higher annual sepsis case volume in the ICU was associated with significantly lower hospital mortality, and this association had no significant interaction with illness severity. A lower volume threshold of 215 treated patients was identified, above which hospital mortality decreased significantly.

**Meaning:**

The findings suggest that patients with sepsis in the UK have higher odds of survival if treated in an ICU with a higher sepsis case volume.

## Teaser

This cohort study assesses the association between the annual volume of patients with sepsis treated in intensive care units of UK hospitals and hospital mortality from sepsis between 2010 and 2016.

## Introduction

Sepsis is a dysregulated host response to infection that results in organ dysfunction.^1^ It is among the leading causes of death worldwide, and the global burden of sepsis is expected to increase as populations age.^2^ The World Health Assembly has urged member states and other stakeholders to strengthen efforts to prevent, diagnose, and treat sepsis.^3^ Patients with sepsis require high-cost interventions in intensive care units (ICUs), where even with prompt treatment, they have a high probability of death.^2^ One strategy to reduce mortality might be to treat patients with sepsis in larger, high-volume ICUs.

Since a seminal report by Luft et al in 1979,^4^ there has been increasing evidence that patients receiving treatment for complex conditions have lower mortality when treated in institutions with a high-volume caseload compared with institutions with a low-volume caseload.^4,5,6,7,8,9,10,11^ Other major benefits are the potential for lower costs by economies of scale and more efficient use of staff and other resources.^12^ The main concerns are the potential for fragmentation of care, the need to transport patients away from their local hospital, and the possibility that high-volume centers will be overwhelmed.^13^

 Sepsis, although common and clinically identifiable, has not attracted much attention in the literature assessing the association of case volume with outcomes.^1,14^ Sepsis requires time-critical interventions provided almost exclusively within the ICU, allowing assessment of the direct association of outcomes with ICU treatment. Thus, we evaluated the association between annual sepsis case volume in an ICU and hospital mortality among patients with sepsis in the UK. We chose mortality as the outcome because sepsis is associated with significant mortality,^15^ and this outcome is not subject to manipulation.

## Methods

### Data

In this cohort study, we analyzed data from the Case Mix Programme database, a national clinical database of all adult patients admitted to ICUs in England, Wales, and Northern Ireland that is coordinated by the Intensive Care National Audit & Research Centre (ICNARC). Details of the validation of the Case Mix Programme database were published previously.^16,17,18,19,20^ Approval for the use of data from the Case Mix Programme was obtained under §251 of the National Health Service Act 2006. The London School of Economics waived the requirement for approval and informed consent because this research involved secondary analysis of an established data set of anonymized data. This study followed the Strengthening the Reporting of Observational Studies in Epidemiology (STROBE) reporting guideline.^21^

### Patient Selection

All admissions of adults with sepsis to 231 general ICUs in England, Wales and Northern Ireland between January 1, 2010, and December 31, 2016, were included. Sepsis cases were identified using the third international consensus definitions for sepsis and septic shock (Sepsis-3).^1^ We considered the index critical care admission for sepsis as an admission for an infection with a sequential organ failure score of 2 or higher. Septic shock was defined as infection with a Sequential Organ Failure Assessment score of 2 or higher with a cardiovascular component or with a serum lactate concentration greater than 18 mg/dL, in accordance with the Sepsis-3 definition.^1^ Patients younger than 16 years, patients for whom all physiological data were missing, and patients who stayed in the ICU for less than 8 hours were excluded.

### Exposure

The exposure was defined as the annual sepsis case volume in an ICU in the year of a patient’s admission; for example, if a patient was admitted to an ICU in 2010, we defined exposure as the sepsis case volume in that ICU in 2010. In the initial data analysis, we followed the common approach of categorizing ICU volumes into quartiles, which we justified given that we were analyzing the complete set of general ICUs in England, Wales, and Northern Ireland. Our preferred approach was to specify volume as a continuous variable and used restricted cubic splines to identify the best-fitting model.

### Study Outcome

The primary outcome was death before discharge from an acute care hospital. Patients who were transferred between ICUs were excluded from the analysis of mortality but included in the estimation of ICU volumes. This was done to avoid confounding results with outcomes from different ICUs. For patients who were readmitted to the ICU, only the first admission was included in the mortality analysis.

### Statistical Analysis

Data were analyzed from January 1, 2010, to December 31, 2016. The risk-adjusted association between ICU volume and acute care hospital mortality was assessed using a mixed-effects logistic model in a 3-level hierarchical structure based on the number of individual patients nested in years nested within ICUs. This mixed-effects approach was used to evaluate the association between the volume of sepsis cases in an ICU and mortality from sepsis while giving adequate control for the within-ICU variation over time. Control variables included in the model were age, sex (with female as the reference), prehospital dependence (with no dependence as the reference), race/ethnicity (with White as the reference), comorbidities (with severe respiratory disease, severe cardiac disease, end-stage kidney disease, severe liver disease, metastatic disease, hematological malignancy, and compromised immune system as the reference), socioeconomic deprivation as measured by the Index of Multiple Deprivation, severity of illness as measured by the ICNARC-2018 score,^22,23^ annual bed occupancy rate, and academic affiliation (with nonuniversity as the reference). Race/ethnicity was defined using the UK Census recommendations of categorization.^24^ Race/ethnicity was included as a control variable because of the association with mortality from sepsis.^25,26,27^ Full details are provided in the eAppendix in the Supplement.

The annual ICU sepsis case volume was initially analyzed as a categorical variable, as in earlier studies.^28^ Categorization is a popular method for studies on case volume and outcomes but has disadvantages. The categories are determined by the distribution of the data; thus, the cutoff points are arbitrary and study specific, limiting generalization. There is also substantial loss of information through categorization, with all ICUs in the same category assumed to have the same mortality risk.

Our subsequent data analysis therefore defined volume as a continuous variable, and we specified restricted cubic splines to allow for assessment of the nonlinear association between case volume and mortality. In making the model more flexible, potential overfitting was avoided, whereas the interpretability of the modeled association was retained. Restricted cubic splines can identify local features and provide stable estimates at the tails of data, making the spline model reliable in identifying a local marginal treatment effect. We fitted models with 3, 4, 5, and 6 knots and used information criteria and likelihood ratio tests to select the model with 3 knots as the most parsimonious.^29^ We used a Wald test to assess the overall association between sepsis case volume and mortality. We specified values of ICU volume at midpoints on the knots to provide a comparison with the quartile model.^30^ Details, including various specification tests, are given in the eAppendix in the Supplement. Significance was defined as *P* < .05 using a 2-tailed test. Data analysis was performed using Stata, version 16.0 (StataCorp LLC).

We used the 3-level hierarchical logistic regression model to account for the clustering of patients within ICUs across years. This approach also estimated random intercepts for each ICU, which were interpreted as the latent ICU-level variation.^31^ Details are included in the eAppendix in the Supplement. We evaluated the significance of the between-ICU variation using a median odds ratio (OR).^32^

#### Subgroup Analysis

We hypothesized that sicker patients would have a lower mortality risk if treated in a high-volume ICU vs a low-volume ICU. To assess this, we performed a test of the interaction between ICU volume and illness severity using the mortality risk estimated using the ICNARC-2018 score. We examined sensitivity in this subgroup analysis by altering the definition of more severely ill. We subsequently defined sicker patients admitted to ICUs as those with septic shock, those with an expected mortality rate greater than 30% as estimated by the ICNARC model, or those who received mechanical ventilation or kidney replacement therapy within 20 hours of ICU admission. Second, we analyzed nonsurgical patients with sepsis to ensure that the observed outcome was not influenced by inclusion of surgical patients with sepsis.

#### Sensitivity Analysis

We used fractional polynomials as an alternate specification of volume as a continuous variable to test the sensitivity of the results to the specification of the association between case volume and outcome.^29^ Fractional polynomials are global functions and may obscure local features, particularly at the tails of the data distribution, and may therefore be less useful than cubic splines in identifying a threshold volume, particularly at low volumes.^33^

We then performed a quantitative bias assessment to assess the influence of unmeasured covariates^34,35^ using E-values. E-values measure the minimum association that an unmeasured covariate would require with both ICU volume and mortality, conditional on the measured covariates, to explain the empirically determined association between case volume and outcome.^35^

In addition, we checked that volume was exogenous. In this study’s model, exogeneity required that ICU volume was not associated with the ICU-level random effect.^36^ Details are provided in the eAppendix in the Supplement.

## Results

### Descriptive Statistics

Of the 305 748 ICU admission episodes (which included readmissions and transfers) meeting the Sepsis-3 criteria between 2010 and 2016, 32 747 (10.7%) were excluded from the mortality analysis. This included 19 809 patients who were readmitted, 12 296 patients transferred between ICUs, and 642 patients who were readmitted and transferred between ICUs. Descriptive statistics for the sample of 273 001 patients with sepsis treated within general ICUs from 2010 to 2016 are shown in Table 1 and eTable 1 in the Supplement; patient flow is shown in eFigure 1 in the Supplement. The median age of the patients was 66 years (interquartile range [IQR], 53-76 years); 148 149 (54.2%) were male, and 248 275 (91.0%) were White. The mean ICNARC-2018 score was 21.0 (95% CI, 20.9-21.0) Most of the patients (80.1%) had no severe medical comorbidity. Of all included patients, 1.8% were recorded as having severe cardiac disease, 4.6% as having severe respiratory disease, 1.9% as having end-stage kidney disease, and 2.2% as having liver disease; 8.8% were recorded as immunocompromised. The mean ICNARC-2018–estimated mortality rate was 29.7% (95% CI, 29.6%-29.8%). Mechanical ventilation was used for 53.1% of patients, 19.9% had a diagnosis of septic shock, and 8.8% had received kidney replacement therapy within 24 hours of ICU admission.

**Table 1.** Characteristics of Patients Admitted to ICUs in the UK Between 2010 and 2016 Across Quartiles of Annual ICU Caseload of Sepsis^a^

Variable	Patients with sepsis^b^					*P* value^c^
	Total (N = 273 001)	Quartile 1 (n = 68 952)	Quartile 2 (n = 69 269)	Quartile 3 (n = 68 289)	Quartile 4 (n = 66 491)	
Age, y						
<54	68 947 (25.2)	17 022 (24.9)	16 550 (24.4)	17 453 (25.5)	17 232 (25.2)	<.001
54-66	69 264 (25.3)	17 322 (25.3)	17 021 (24.9)	17 110 (25.0)	17 007 (24.8)	
67-76	68 289 (25.0)	18 011 (25.4)	18 256 (25.8)	17 674 (25.0)	16 894 (23.9)	
>76	66 491 (24.4)	16 592 (25.4)	17 337 (26.5)	16 052 (24.6)	15 358 (23.5)	
Sex						
Male	148 149 (54.2)	37 226 (54.0)	37 326 (53.9)	37 280 (54.6)	36 317 (54.6)	.006
Female	124 852 (45.7)	31 726 (46.0)	31 943 (46.1)	31 009 (45.4)	30 174 (45.4)	
Race/ethnicity						
White	248 275 (91.0)	63 059 (91.5)	64 504 (93.2)	62 712 (91.9)	58 000 (87.2)	<.001
Asian	9438 (3.5)	2472 (3.6)	1779 (2.6)	2114 (3.1)	3073 (4.6)	
Black	5504 (2.0)	1304 (1.9)	1092 (1.6)	1036 (1.5)	2072 (3.1)	
Mixed or other^d^	9617 (3.5)	2070 (3.0)	1848 (2.7)	2353 (3.4)	3346 (5.0)	
Comorbidities						
Cardiac	4857 (1.8)	1390 (2.0)	1032 (1.5)	11 318 (1.9)	1117 (1.7)	<.001
Respiratory	12 498 (4.6)	3187 (4.6)	2870 (4.2)	2863 (4.2)	3578 (5.4)	<.001
ESKD	5171 (1.9)	1002 (1.5)	967 (1.4)	1297 (1.9)	1905 (2.9)	<.001
Liver	6030 (2.2)	1208 (1.8)	1285 (1.9)	1468 (2.2)	2069 (3.1)	<.001
Metastatic cancer	6598 (2.4)	1610 (2.4)	1509 (2.2)	1709 (2.5)	1770 (2.7)	<.001
Hematologic cancer	9763 (3.6)	2349 (3.4)	2178 (3.2)	2551 (3.8)	2685 (4.1)	<.001
Immunocompromised	24 035 (8.8)	5884 (8.6)	5553 (8.1)	6287 (9.3)	6311 (9.5)	<.001
Level of dependency before acute care hospitalization						
Independent	184 850 (68.0)	47 150 (68.7)	47 545 (68.9)	44 925 (66.1)	45 230 (68.3)	<.001
Some assistance	81 913 (30.1)	20 220 (29.5)	20 233 (29.3)	21 851 (32.1)	19 609 (29.6)	
Total dependence	5071 (1.9)	1262 (1.8)	1223 (1.8)	1214 (1.8)	1372 (2.1)	
Usual residence before hospitalization						
Home	264 730 (97.0)	66 816 (96.9)	67 200 (97.0)	66 286 (97.1)	64 428 (96.9)	<.001
Work or non–health-related institution	564 (0.2)	144 (0.2)	132 (0.2)	143 (0.2)	145 (0.2)	
Nursing home, hospice, or health-related institution	6756 (2.5)	1781 (2.6)	1716 (2.5)	1646 (2.4)	1613 (2.4)	
No fixed address	951 (0.4)	221 (0.3)	221 (0.3)	214 (0.3)	305 (0.5)	
IMD quintile						
1	69 728 (25.7)	15 507 (22.7)	15 654 (22.7)	17 144 (25.3)	21 423 (32.5)	<.001
2	58 496 (21.6)	15 047 (22.0)	15 574 (22.6)	13 632 (20.1)	14 243 (21.6)	
3	53 199 (19.6)	14 075 (20.6)	14 345 (20.8)	13 200 (19.4)	11 579 (17.5)	
4	47 306 (17.5)	12 864 (18.8)	12 623 (18.3)	12 095 (17.8)	9724 (14.7)	
5	42 400 (15.6)	10 776 (15.8)	10 743 (15.6)	111 833 (17.4)	9048 (13.7)	
Admission type						
Medical	204 524 (74.9)	52 890 (76.7)	51 067 (73.7)	50 163 (73.4)	50 404 (74.9)	<.001
Elective surgery	11 780 (4.3)	3167 (4.6)	2825 (4.1)	2710 (4.0)	3078 (4.6)	
Emergency surgery	56 671 (20.8)	12 886 (18.7)	15 368 (22.2)	15 409 (22.6)	13 008 (19.6)	
APACHE II score, mean (95% CI)	18.4 (18.4-18.4)	18.5 (18.4-18.5)	18.3 (18.2-18.3)	18.5 (18.4-18.5)	18.5 (18.5-18.6)	<.001
ICNARC score, mean (95% CI)	21.0 (20.9-21.0)	21.3 (21.2-21.4)	21.1 (21.0-21.1)	20.9 (20.9-21.0)	20.4 (20.4-20.5)	<.001
ICNARC estimated probability of death, mean (95% CI), %	29.7 (29.6-29.8)	30.7 (30.5-30.9)	29.8 (29.6-30.0)	29.5 (29.3-29.7)	28.8 (28.6-29.0)	<.001
Kidney failure in the first 24 h	23 573 (8.8)	6154 (9.1)	6253 (9.2)	5866 (8.7)	5300 (8.1)	<.001
Mechanical ventilation	145 041 (53.1)	38 278 (55.5)	36 994 (53.4)	36 035 (52.8)	33 734 (50.7)	<.001
Septic shock	54 419 (19.9)	14 458 (21.0)	13 912 (20.1)	13 016 (19.1)	13 033 (19.6)	<.001
ICU length of stay, median (IQR), h	90 (42-189)	93 (41-200)	88 (41-186)	90 (42-187)	88 (42-186)	<.001
Hospital length of stay, median (IQR), d	14 (7-28)	14 (7-29)	14 (7-27)	14 (7-28)	15 (7-30)	<.001
ICU mortality	62 277 (22.8)	16 156 (23.4)	16 245 (23.5)	15 567 (22.8)	14 309 (21.5)	<.001
Hospital mortality	86 728 (31.9)	22 789 (33.3)	22 381 (32.5)	21 263 (31.3)	20 295 (30.7)	<.001

Abbreviations: APACHE II, Acute Physiologic Assessment and Chronic Health Evaluation II; ESKD, end-stage kidney disease; ICNARC, Intensive Care National Audit & Research Centre; ICU, intensive care unit; IMD, Index of Multiple Deprivation; IQR, interquartile range.

^a^ Quartile 1 was 12 to 177 cases; quartile 2, 178 to 242 cases; quartile 3, 243 to 334 cases; and quartile 4, 335 to 744 cases.

^b^ Data are presented as number (percentage) of patients unless otherwise indicated. Data were missing for some patients in certain categories, so numbers may not sum to the totals. Percentages may not sum to 100 owing to rounding.

^c^ For categorical variables, a χ^2^ test was used. For continuous variables, analysis of variance was used to analyze the differences in means between groups.

^d^ Mixed included mixed White and Black Caribbean, mixed White and Black African, mixed White and Asian, and any other mixed race/ethnicity; other included any ethnic group not stated.

The unadjusted hospital mortality rate was 31.9% (95% CI, 31.8%-32.1%). Hospital mortality was 33.3% in the lowest volume quartile compared with 30.7% in the highest quartile (Table 1).

Of the 231 ICUs, 122 (52.8%) were in non–university hospitals, 39 (16.9%) were university affiliated, and 70 (30.3%) were university based. The median number of ICU beds was 8 (IQR, 6-10) in the lowest quartile of ICU volume compared with 23 (IQR, 18-28) in the highest quartile (Table 2 and eTable 2 and eFigure 2 in the Supplement).

**Table 2.** Characteristics of 231 ICUs Across Quartiles of Annual Sepsis Volume^a^

Variable	Median (IQR)					*P* value^b^
	Total	Quartile 1	Quartile 2	Quartile 3	Quartile 4	
ICU beds	13 (9-18)	8 (6-10)	11 (9-13)	15 (12-17)	23 (18-28)	<.001
Occupancy, %	73.5 (67.9-79.5)	67.5 (59.4-74.2)	71.7 (66.9-77.0)	74.0 (70.5-79.6)	78.6 (74.8-82.9)	<.001
Sepsis volume	242 (177-334)	136 (112-160)	214 (197-228)	280 (260-302)	415 (378-483)	<.001
Nonsepsis volume	497 (346-747)	288 (220-369)	432 (343-521)	572 (461-706)	918 (713-1176)	<.001
Total volume	742 (533-1087)	427 (353-516)	646 (552-737)	856 (732-997)	1348 (1173-1614)	<.001

Abbreviations: ICU, intensive care unit; IQR, interquartile range.

^a^ Quartile 1 was 12 to 177 cases; quartile 2, 178 to 242 cases; quartile 3, 243 to 334 cases; and quartile 4, 335 to 744 cases.

^b^ For categorical variables, a χ^2^ test was used. For continuous variables, analysis of variance was used to analyze the differences in means between groups.

### Regression Analysis

**Figure 1.**
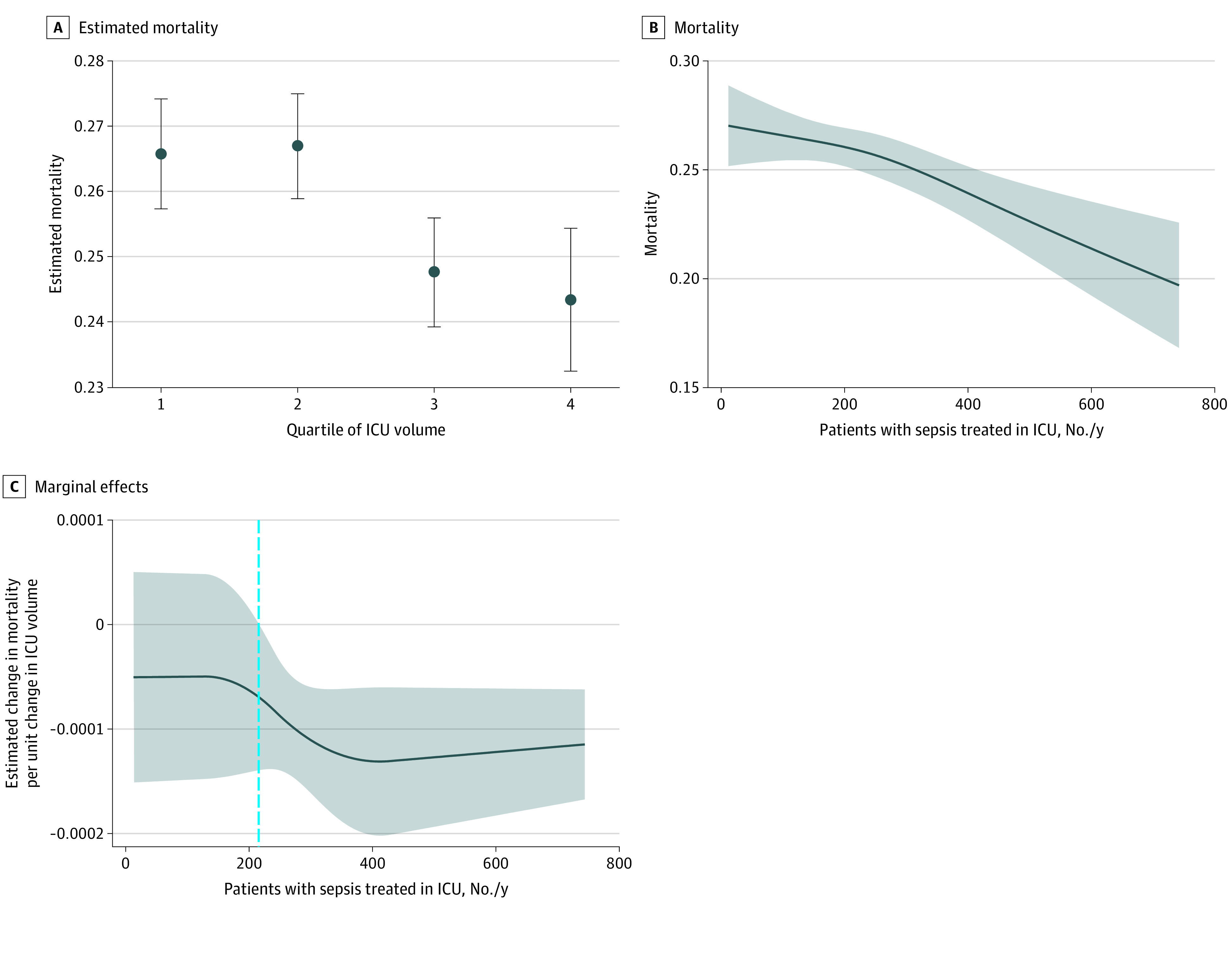
Association Between Sepsis Case Volume in an Intensive Care Unit (ICU) and Acute Care Hospital Mortality A, Markers represent adjusted probabilities and whiskers indicate 95% CIs. B, Shaded area indicates 95% CI. C, Shaded area indicates 95% CI; dashed vertical line indicates the threshold at which an increase in volume resulted in a significant reduction in estimated mortality.

The logistic regression model revealed a statistically significant reduction in hospital mortality among patients admitted to ICUs in the highest quartile of sepsis volume compared with those admitted to ICUs in the lowest quartile (OR, 0.89; 95% CI, 0.82-0.96; *P* = .002) (Table 3, Figure 1, and eFigure 3 in the Supplement).

**Table 3.** Odds Ratios of Acute Hospital Mortality Specifying Intensive Care Unit Volume as Categorial and Using Restricted Cubic Splines

Model	OR (95% CI)	*P* value
Categorical^a^		
Quartile 1	1 [Reference]	NA
Quartile 2	1.01 (0.96-1.05)	.80
Quartile 3	0.91 (0.86-0.96)	.001
Quartile 4	0.89 (0.82-0.96)	.002
Restricted cubic splines^b^		
Midpoint origin and knot 1 (n = 63)	1 [Reference]	NA
Midpoint knot 1 and knot 2 (n = 184)	0.97 (0.91-1.03)	NA
Midpoint knot 2 and knot 3 (n = 335)	0.90 (0.82-0.99)	NA
Midpoint knot 3 and maximum (n = 589)	0.75 (0.66-0.86)	NA

Abbreviations: NA, not applicable; OR, odds ratio.

^a^ Quartile 1 was 12 to 177 cases; quartile 2, 178 to 242 cases; quartile 3, 243 to 334 cases; and quartile 4, 335 to 744 cases.

^b^ Per 50 patients with sepsis.

With volume modeled as a restricted cubic spline, greater ICU volume was associated with lower hospital mortality (Figure 1 and eTable 3 in the Supplement). The marginal treatment effect refers to the estimated change in mortality per unit change in ICU volume and varied with the point of estimation in nonlinear models. The restricted cubic spline specification identified a lower threshold of 215 patients with sepsis treated per year, above which greater sepsis case volume in the ICU was associated with lower mortality (Figure 1). Above this volume threshold, there was a significant reduction in mortality (Figure 1 and Table 3). Altogether, 38.8% of patients with sepsis were treated in ICUs below this threshold value. We could not identify an upper threshold value.

The between-ICU practice variation was derived from the mixed-effects model using estimated random intercepts as a measure of latent quality. The median OR for hospital mortality was 1.27 (95% CI, 1.23-1.30), suggesting significant unexplained between-ICU practice variation. The variance within the same ICU across the study period did not change significantly, suggesting that an individual ICU’s performance in terms of mortality was stable over time (eFigure 4 in the Supplement).

#### Subgroup Analyses

**Figure 2.**
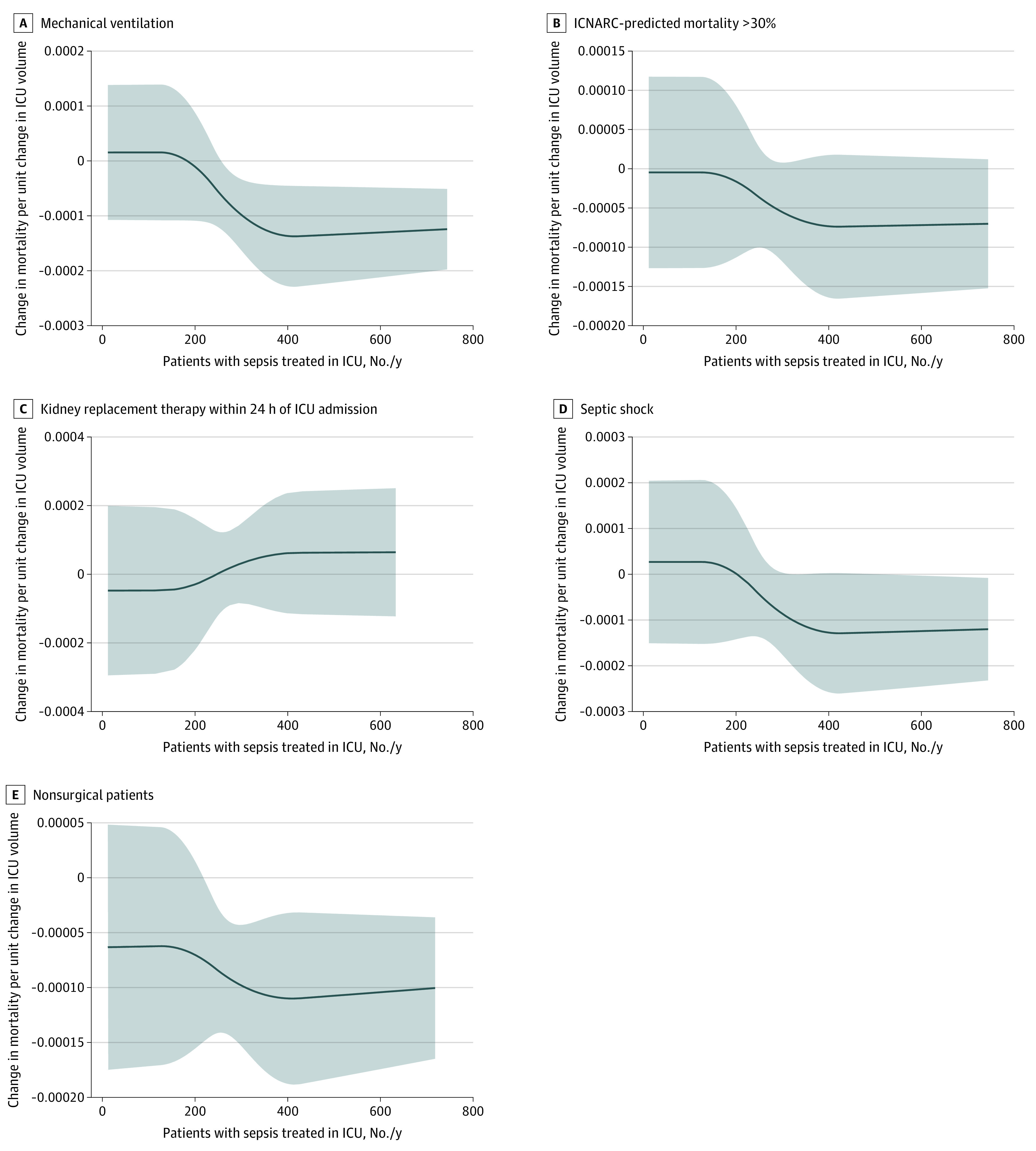
Subgroup Analysis of Marginal Treatment Effects of Intensive Care Unit (ICU) Volume Shaded areas indicate 95% CIs. ICNARC, Intensive Care National Audit & Research Centre.

There was no significant interaction between ICU volume and severity of illness as described by the ICNARC-2018 score (β [SE], –0.00014 [0.00024]; *P* = .57). In addition, subgroup analyses of patients defined as severely ill also did not identify a lower sepsis case volume threshold for mortality (subgroup receiving mechanical ventilation: β [SE], –0.00056 [0.00019]; *P* = .003; subgroup with >30% predicted mortality: β [SE], –0.00032 [0.00019]; *P* = .10; subgroup receiving kidney replacement therapy <24 hours after ICU admission: β [SE], 0.00023 [0.00035]; *P* = .51; and subgroup with septic shock: β [SE], –0.00051 [0.00026]; *P* = .052) (Figure 2). The association between case volume and mortality found in the subgroup of nonsurgical patients with sepsis was similar to that in the entire cohort (β [SE], 0.00053 [0.00018]; *P* = .002) (Figure 2 and eFigure 5 in the Supplement).

#### Sensitivity Analysis

The association between case volume and mortality remained statistically significant in the fractional polynomial model (eFigure 6 and eTables 4 and 5 in the Supplement). The quantitative bias analysis returned an E-value of 1.31 (lower bound of 95% CI, 1.17) (eFigure 7 and eAppendix in the Supplement). The lack of statistical significance in the between- and within-cluster effects for ICU volume indicated a lack of correlation in the ICU volume and the ICU random effect, in support of the assumption that ICU sepsis volume is exogenous (eTable 6 in the Supplement).

## Discussion

In this cohort study, we found a significant association between the sepsis case volume in an ICU and hospital mortality from sepsis, and the association was consistent across the categorical and nonlinear specifications of ICU volume. The sepsis volumes included in this study exceeded the spectrum of volumes described in other published studies,^37,38^ thereby improving the power to detect even a small association between case volume and mortality.

The study also identified a lower volume threshold of 215 patients treated per year, above which there was a statistically significant reduction in mortality. This threshold was estimated based on our preferred empirical specification using a 3-knot restricted cubic spline regression, which also controlled for a rich set of covariates to model the association between the case volume and mortality. There was no significant interaction between case volume and severity of illness. The study found that significant ICU practice variation was not explained by patient or hospital characteristics, implying that sample selection was not distorting the associations described. The within-ICU variation remained unchanged across years, suggesting that higher-performing ICUs maintained good performance over time.

The study’s findings are based on a large population of ICUs observed over time. A recent meta-analysis^28^ of smaller observational studies found an overall positive association of outcome with ICU volume; however, there was significant heterogeneity. Some previous studies^39,40^ that did not account for the clustered nature of the data revealed upwardly biased estimates of the association between case volume and outcome. The hierarchical structure of the current data analysis may account for the more modest association found in this study compared with other published studies.^28^

Prior studies^28,37,41,42^ of the association between case volume and outcomes among patients with sepsis have shown conflicting results. The literature is subject to limitations.^37,41,43,44,45^ First, many of the studies^38,46,47,48,49,50^ of case volume and outcomes among patients with sepsis were undertaken in the US, where there is a complex system of health care funding and where the observed benefits attributed to volume may to some extent reflect unmeasured disparities in access to care as well as socioeconomic disparities. Studies undertaken in countries such as Canada, Finland, or the UK, where there are single-payer, publicly funded health care systems, have not shown a consistent association between the volume of sepsis cases and outcomes.^37,45,51^ Second, in comparisons between high- and low-volume specialist and nonspecialist services, some of the observed benefits of high case volume may in fact be a result of specialization.

Third, a major limitation of the existing literature on the association between ICU sepsis case volume and outcomes is the lack of a criterion standard for defining volume.^28^ Examining quartiles does not improve the general understanding of the association between sepsis case volume in the ICU and outcomes because ICUs considered to be high volume in 1 study may be within a lower volume quartile in another study because the quartiles are specific to each data set. In this study, we used restricted cubic splines that allowed flexibility in describing the functional form of volume in regression models. In using the full range of data, these methods provided a more accurate description of the association between volume and mortality, with the additional ability to suggest optimal volume thresholds. Fourth, many studies included a small number of ICUs with a narrow spectrum of volumes, leaving them underpowered to detect a small but statistically and clinically meaningful association between case volume and outcome.

In addition, most studies use secondary administrative data collected for other uses. Such data have inherent limitations in both the identification of sepsis and the characteristics of patients and ICUs. This study used a large clinical database of patients with sepsis admitted to all general ICUs in the UK, allowing us to perform detailed risk adjustment and identify ICU-specific characteristics.

In the UK, ICUs are unable to make a risk-based selection of patients with sepsis who are at low risk of mortality because sepsis is an emergency condition and patients are taken to the nearest hospital, often by the ambulance service. The empirical findings of this study suggest treatment benefits could be made through a concentration of ICU facilities, similar to the successful policy adopted by the National Health Service in some areas with respect to the treatment of stroke.^52^

### Strengths and Limitations

This study has strengths. In terms of completeness, coverage, and representativeness of the data, this was one of the largest studies to examine the association between ICU volume and outcomes for patients with sepsis. By including all general ICUs in England, Wales, and Northern Ireland, the study assessed the entire adult population treated for sepsis in these countries during the study period.^16^ This study used a granular clinical database with a standardized data collection process and a validated risk adjustment model developed for UK ICUs, and it used the international consensus Sepsis-3 definition to identify patients with sepsis.^1,16,22,38^ The potential for selection bias was limited by using a cohort of patients with sepsis treated in publicly funded general ICUs within the UK National Health Service, which covers the whole population.

This study also has limitations. We used observational data that may have been subject to unmeasured confounding. We evaluated the potential for unmeasured confounding using E-values,^35^ which resulted in a threshold risk ratio of 1.17. Although the E-value is modest, we believe that, given the detailed clinical data recorded in the Case Mix Programme database, substantial unmeasured confounding was improbable. If an omitted variable was associated with an included covariate, the omitted variable would not result in substantial bias. The E-value assumes that the distribution of unmeasured confounders is as unfavorable as possible and represents the most conservative scenario.^35^

As is typical in the literature on case volume and outcome, we used the contemporaneous ICU volume as the exposure. This did not distinguish between the static scale effects of volume and the cumulative learning-by-doing effects. In addition, the data set did not have details on processes of care specific to sepsis, such as timing of the first dose of antibiotics. We were therefore unable to establish the underlying mechanism of association of sepsis case volume in the ICU with mortality from sepsis.

## Conclusions

In this cohort study, sepsis case volume in an ICU was significantly associated with hospital mortality from sepsis, and a volume threshold associated with an improvement in mortality was identified. Further research is required to better understand the mechanism of this association.

## Notes

Supplement. **eAppendix.** Supplementary appendix **eFigure 1.** Study patient flow **eFigure 2.** Violin plot of annual ICU sepsis volume between 2010 and 2016 **eTable 1.** Characteristics of patients admitted to the ICU with between 2010 and 2016 and quantiles of annual ICU case load of sepsis **eTable 2.** ICU characteristics between 2010 and 2016 **eFigure 3.** Results of the regression analysis with volume as quartiles **eTable 3.** Testing the statistical significance between models of increasing complexity with volume defined as a restricted cubic spline **eFigure 4.** Variation within ICUs across years empirical Bayes predication **eFigure 5.** Subgroups analysis: Adjusted probability of mortality **eTable 4.** Comparisons of model fit for fractional polynomial **eFigure 6.** Functional form of fractional polynomial **eTable 5.** A comparison of the information criteria for the linear, fractional polynomial and restricted cubic spline models **eFigure 7.** Value of the joint minimum strength of association that an unmeasured confounder must have with both an increase in ICU sepsis volume and acute hospital mortality to explain away the volume outcome relationship, expressed as a risk ratio **eTable 6.** Within and between cluster effects of ICU volume test for exogeneity eReferences
