# Supplementary material for: Association of Annual Intensive Care Unit Sepsis Caseload With Hospital Mortality From Sepsis in the United Kingdom, 2010-2016
Source: JAMA Netw Open. 2021 Jun 29;4(6):e2115305. doi: 10.1001/jamanetworkopen.2021.15305 (PMC8243236; doi:10.1001/jamanetworkopen.2021.15305)
Supplement: Supplement. — eAppendix. Supplementary appendix eFigure 1. Study patient flow eFigure 2. Violin plot of annual ICU sepsis volume between 2010 and 2016 eTable 1. Characteristics of patients admitted to the ICU with between 2010 and 2016 and quantiles of annual ICU case load of sepsis eTable 2. ICU characteristics between 2010 and 2016 eFigure 3. Results of the regression analysis with volume as quartiles eTable 3. Testing the statistical significance between models of increasing complexity with volume defined as a restricted cubic spline eFigure 4. Variation within ICUs across years empirical Bayes predication eFigure 5. Subgroups analysis: Adjusted probability of mortality eTable 4. Comparisons of model fit for fractional polynomial eFigure 6. Functional form of fractional polynomial eTable 5. A comparison of the information criteria for the linear, fractional polynomial and restricted cubic spline models eFigure 7. Value of the joint minimum strength of association that an unmeasured confounder must have with both an increase in ICU sepsis volume and acute hospital mortality to explain away the volume outcome relationship, expressed as a risk ratio eTable 6. Within and between cluster effects of ICU volume test for exogeneity eReferences [file jamanetwopen-e2115305-s001.pdf]

Supplementary Online Content

Maharaj R, McGuire A, Street A. Association of annual intensive care unit sepsis caseload with hospital mortality from sepsis in the United Kingdom, 2010-2016. *JAMA Netw Open*. 2021;4(6):e2115305. doi:10.1001/jamanetworkopen.2021.15305

eAppendix. Supplementary appendix
eFigure 1. Study patient flow
eFigure 2. Violin plot of annual ICU sepsis volume between 2010 and 2016
eTable 1. Characteristics of patients admitted to the ICU with between 2010 and 2016 and quantiles of annual ICU case load of sepsis
eTable 2. ICU characteristics between 2010 and 2016
eFigure 3. Results of the regression analysis with volume as quartiles
eTable 3. Testing the statistical significance between models of increasing complexity with volume defined as a restricted cubic spline
eFigure 4. Variation within ICUs across years empirical Bayes predication
eFigure 5. Subgroups analysis: Adjusted probability of mortality
eTable 4. Comparisons of model fit for fractional polynomial
eFigure 6. Functional form of fractional polynomial
eTable 5. A comparison of the information criteria for the linear, fractional polynomial and restricted cubic spline models
eFigure 7. Value of the joint minimum strength of association that an unmeasured confounder must have with both an increase in ICU sepsis volume and acute hospital mortality to explain away the volume outcome relationship, expressed as a risk ratio
eTable 6. Within and between cluster effects of ICU volume test for exogeneity
eReferences

This supplementary material has been provided by the authors to give readers additional information about their work.

© 2021 Maharaj R et al. *JAMA Network Open*.

eAppendix

Table of contents

1. Statistical methods
    a. Multilevel model
        i. Rationale
        ii. Empirical model
            ii.a Quartiles
            ii.b Restricted cubic splines
        iii. Fixed versus random effects
        iv. Interpretation of the random effects
    b. Control variables
2. Descriptive variables
    a. Patient variables
    b. ICU variables
3. Additional descriptive data
    a. eFigure 1. Study patient flow
    b. eFigure 2. Violin plot of annual ICU sepsis volume between 2010 and 2016
    c. eTable1. Characteristics of patients admitted to the ICU with between 2010 and 2016 and quantiles of annual ICU case load of sepsis.
    d. eTable. 2 ICU characteristics between 2010 and 2016
4. Additional results
    a. eFigure 3. Results of the regression analysis with volume as quartiles

© 2021 Maharaj R et al. *JAMA Network Open*.

b. eTable 3. Testing the statistical significance between models of increasing complexity with volume defined as a restricted cubic spline
c. eFigure 4. Variation within ICUs across years empirical Bayes predication
d. eFigure 5. Subgroups analysis: Adjusted probability of mortality.

5. Sensitivity analyses and robustness checks
   a. Fractional polynomial and selection procedure
      i. eTable 4. Comparisons of model fit for fractional polynomial
      ii. eFigure 6. Functional form of fractional polynomial
      iii. eTable 5. A comparison of the information criteria for the linear, fractional polynomial and restricted cubic spline models.
   b. E-values
      i. Rationale
      ii. Figure 7. Value of the joint minimum strength of association that an unmeasured confounder must have with both an increase in ICU sepsis volume and acute hospital mortality to explain away the volume outcome relationship
   c. Checking for exogeneity of ICU volume
      i. Rationale
      ii. eTable 6. Within and between cluster effects of ICU volume test for exogeneity

© 2021 Maharaj R et al. *JAMA Network Open*.

eAppendix

The supplementary appendix is set out as follows: We first introduce the hierarchical structure of the data and the multilevel model. We then explore the specification of ICU sepsis volume as quartiles and then as a restricted cubic spline. We perform a sensitivity analysis of specifying volume as a fractional polynomial which is described later in the appendix. We explain the motivation for using a random effects model compared with a fixed effects model and describe the use of the associated empirical Bayes method to predict the ICU-level effect using the estimated random effects. The control variables used in the analysis are then described. We conduct a sensitivity analysis using E-values to explore the potential effects of any missing control variables which is described later in the appendix. We provide additional descriptive data and then return to the results of the empirical analysis and sensitivity analyses. To check the robustness of the random effects model we undertook a regression-based alternative to the Hausman test for endogeneity. We provide a more detailed rationale for the use of the random effects model and conclude with the results of the robustness check.

© 2021 Maharaj R et al. *JAMA Network Open*.

1. Statistical Methods

a) Multilevel models

i. Rationale

The data used in this study feature patients nested within years nested within ICUs, thereby forming a natural hierarchical structure that is suited to a multilevel modelling technique that models each level of the structure simultaneously. Patients treated in the same year, in the same ICU are more likely to have similarities than patients across different years and different ICUs. Multilevel models have been widely used in education for examining the performance across schools[1]. The use of multilevel modelling in health services research has also grown with the increasing availability of patient level data. A landmark paper by Goldstein and Spiegelhalter argued in favour of using the empirical Bayes analysis, which can be related to multilevel modelling in terms of sequential analysis of variation, to make institutional comparisons in terms of utilisation and mortality across health care settings[2].

ii. Empirical model

ii.a ICU volume as quartiles

As it is used commonly in the literature, we first consider volume in terms of quartiles. To account for the multi-level structure of the data, a 3-level random intercept model is specified as follows:

$$logit = \left\{Pr\left(y_{ijt} = 1 | x_{ijt}, u_{jt}^{(2)}, u_j^{(3)}\right)\right\}$$

$$= \beta_0 + \beta_1 ICU\ VOLUME\_Q2_{\ ijt} + \beta_2 ICU\ VOLUME\ \_Q3_{ijt}$$

$$+ \beta_4 ICU\ VOLUME\ \_Q4_{ijt} + \beta'\Omega'_{ijt} + \beta'\Phi_j + u_{jt}^{(2)} + u_j^{(3)}$$

© 2021 Maharaj R et al. *JAMA Network Open*.

In this model y_{ijt} is the outcome variable, within acute hospital mortality and ICU volume is categorised into quartiles using quartile 1 ($ICU\,VOLUME\_Q1$) as the reference category. Here Ω'_{ijt} is a vector of all patient- level covariates, Φ'_{j} is a vector for time invariant ICU-level covariates, $u_{jt}^{(2)} \sim N(0, \psi^{(2)}$ is the random intercept for year within the ICU and $u_{j}^{(3)} \sim N(0, \psi^{(3)})$ is the random intercept varying over ICUs. The random intercept $u_{jt}^{(2)}$ and $u_{j}^{(3)}$ are assumed to be independent of any covariates (exogenous).

ii.b ICU volume as restricted cubic splines

Categorisation of ICU volume into quartiles assumes that all values within the quartile have the same relationship with mortality. An alternative modelling strategy is to allow for a non-linear effect of ICU volume on mortality. To do this we fitted a restricted cubic splines of sepsis volume and assessed the model with a likelihood ratio test and information criteria. Cubic splines are defined as piecewise-polynomial line segments across the distribution of the ICU volume variable, x [3]. The splines are polynomials within intervals of the volume variable, that connect each segment of the distribution. A linear spline is a set of line segments that divides the ICU volume at intervals a, b and c referred to as knots (k). Cubic polynomials allow for fitting of non-linear curves. The cubic splines are made to join at the knots by restricting the first and second derivative of the function to agree at the knots (i.e., there should be no gap in the spline curve). The restricted cubic spline has the further restriction of being linear before the first knot and after the last knot. The regression coefficients determine the shape of the curve and are considered shape parameters. Standard statistical tests can determine if the coefficients are equal to zero i.e., whether or not there is an association between volume and mortality. It is usual to present the results of restricted cubic splines

© 2021 Maharaj R et al. *JAMA Network Open*.

graphically with confidence intervals [4]. There are several ways to compare model fit. Unlike fractional polynomials, there is no formal selection procedure for deciding on the best fitting restricted cubic spline model. We used information criteria (AIC and BIC) to compare non-nested models and likelihood ratio tests to compare nested models. All such tests lend support to our preferred specification A further specification of volume as a fractional polynomial is included as a sensitivity analysis.

iii. Fixed versus random effects

The choice between fixed and random effects specification should be based on the perspective of the investigator. Random effects assume the ICUs are a sample population randomly drawn from a common population, with the inference given to any omitted variables assumed to be related to the whole population. Fixed effects draw inference purely from the effects analysed within the sample. Our interest lies in making inference which supports generalisation to the underlying population of ICUs, and our preferred specification is a random effect one. We do test this choice through application of a Hausman test for misspecification: essentially testing whether the differences in individual effect can be attributed to *chance. The results* are included in eTable 6 and confirm the appropriateness of using a random effects model.

Time fixed effects would capture all unmeasured ICU or patient characteristics within a year. This assumes that there is between-ICU correlation within year.

© 2021 Maharaj R et al. *JAMA Network Open*.

Instead, the time random effects allow for correlation for the same ICU across years. A reason for this might be that the management of sepsis within each ICU is likely to be more similar over time than management across ICUs in the same time period. Hence, we believe that use of random effects to be a less restrictive and closer to reality than using fixed effects.

iv. Interpretation of the random effects

Random effects can be related to the shrinkage estimates in the empirical Bayes literture, as if there is small random intercept variance it can be thought of as an informative prior. Of course this could be counterbalanced by a large level-1 residual variance which would represent uniformative data or a small (ICU) cluster size which would reflect an uninformative cluster.

b) Control variables

To control for confounding factors the models take into account various patient-level and ICU level characteristics. We used E-values to explore the potential effect of missing control variables. This is described in more detail as a sensitivity analysis later in the appendix.

2. Descriptive statistics

a. Patient characteristics

We categorise age into quartiles and include dummy variables for gender. Ethnicity is categorised into White, Asian, Black and Mixed/Other. Co-morbidities are a set of dummy

© 2021 Maharaj R et al. *JAMA Network Open*.

variables for the presence of very severe cardiovascular disease, severe respiratory disease, end stage kidney disease, severe liver disease, metastatic cancer and haematological malignancy. Level of dependency was categorised as being fully independent, requiring some assistance or being fully dependant on assistance. Usual residence prior to hospitalisation was categorised into home, a non-health related institution, a health-related institution such as a nursing home or hospice and no fixed address. Homelessness and residence in a health care institution prior to hospitalisation has been associated with higher mortality from sepsis even after adjustment for comorbidities and disease severity[5, 6]. Socioeconomic status was described using the Index of Multiple Deprivation (2011), categorized into quintiles. The IMD measure relative levels of deprivation in 32,844 small areas or neighbourhoods in England, by combining information about the residents´ income, employment, education, skills and training, health and disability, crime, barriers to housing and services, and environmental conditions. We categorise the index into quintiles, with IMD=1 indicating the most deprived neighbourhoods[7].

The type of admission was categorised as medical, elective surgery and emergency surgery. The severity of illness was quantified by the APACHE II and ICNARC scores. The APACHE II (Acute Physiology, Age, Chronic Health Evaluation) model is a severity of critical illness model developed in 1985 [8] . The score ranges from 0 to 71, with a higher score predicting a higher mortality. The ICNARC score was first published in 2007 and then recalibrated in 2014, 2015 and in 2018[9]. The score is from 0 to 100 based on weightings for deviations from normal in the twelve physiological parameters during the first 24 hours in the ICU as well as additional weights from age, indication for admission, surgical urgency, source of admission and cardiopulmonary resuscitation prior to admission[9-11]. The type of treatment received in

© 2021 Maharaj R et al. *JAMA Network Open*.

the ICU include renal replacement therapy, mechanical ventilation, and circulatory support. Septic shock is a high severity sub-category of sepsis with an expected unadjusted mortality of about 41%[12]. ICU length of stay is described in hours. Hospital length of stay is described in days and includes the days of hospitalisation prior to ICU admission.

b. ICU characteristics

ICU specific characteristics include the academic affiliation, number of ICU beds, annual bed occupancy, sepsis caseload and total caseload.

© 2021 Maharaj R et al. *JAMA Network Open*.

3. **Additional data**

a. **eFigure 1. Study patient flow**

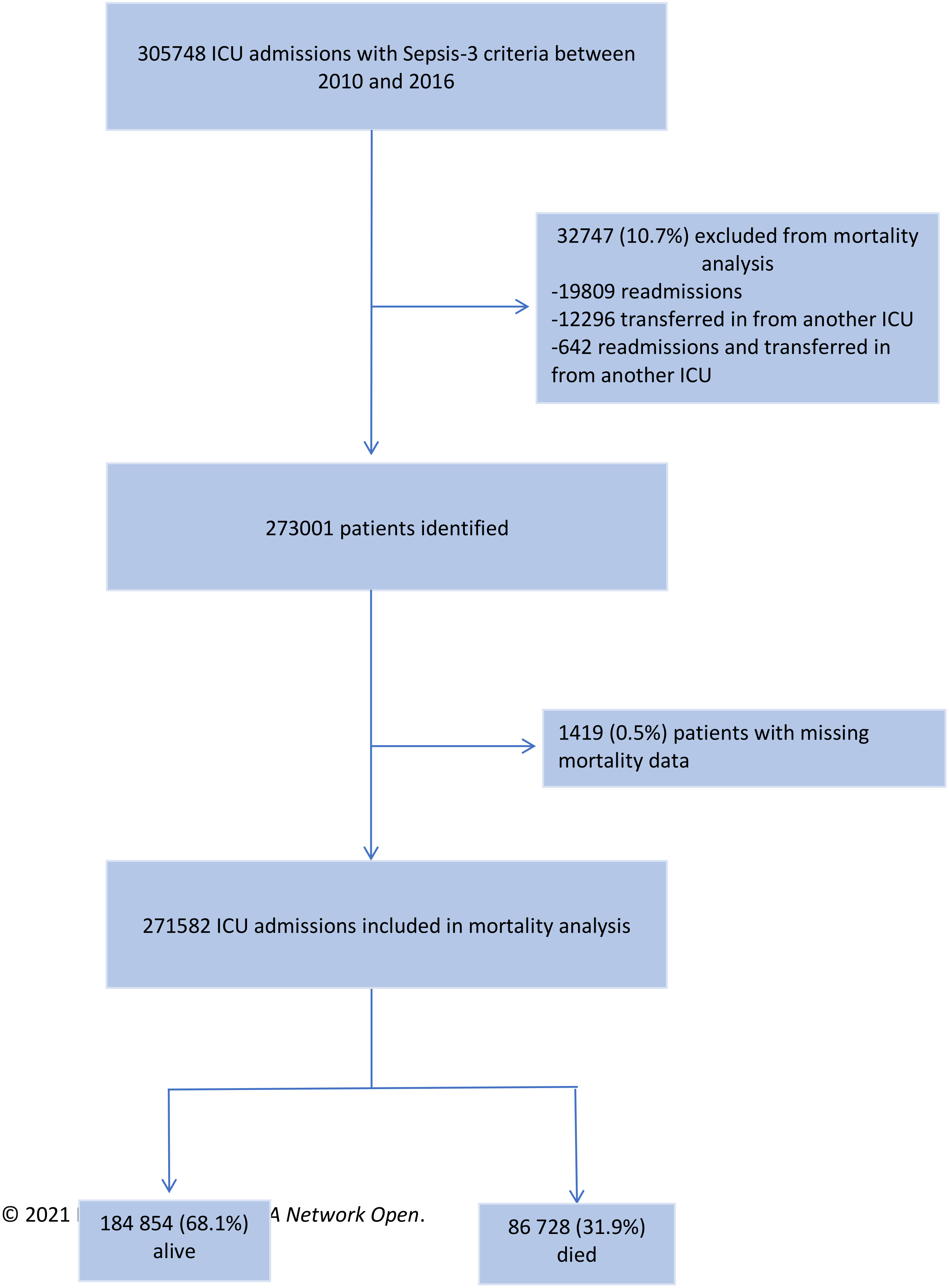

© 2021 [illegible]A *Network Open*.

b. eFigure 2. Violin plot of annual ICU volume and annual ICU sepsis volume between 2010 and 2016

Violin plots are a method for displaying the distribution of the data, combining a boxplot and a kernel density plot. The information contained is the median (white dot), the interquartile range (bar in the centre of the violin). The wider areas of the violin correspond to increased frequency at that y-value. The thin lines that extend from the bar are the lower and upper adjacent values, defined as first quartile -1.5 IQR and third quartile +1.5 IQR. The median total annual ICU volume increased from a caseload of 666 [IQR 459-911) in 2010 to a caseload of 827 [IQR 598-1288] in 2016. The median annual sepsis volume increased from 213 [IQR 156-274] in 2010 to 276 [IQR 200-382].

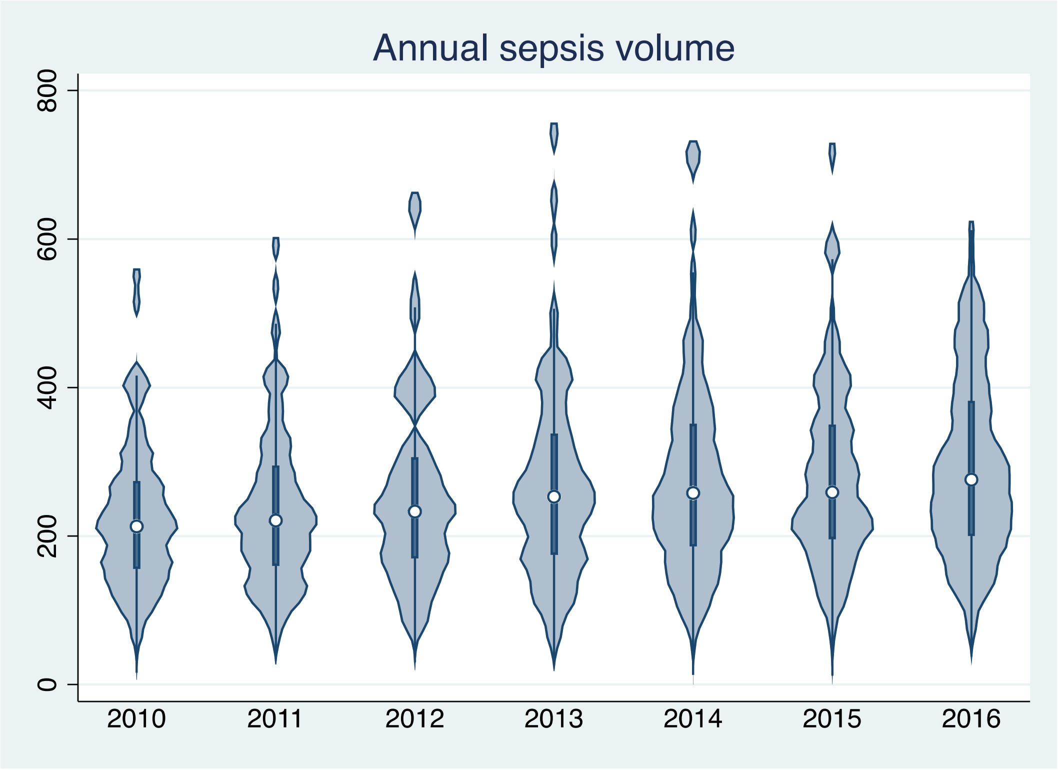

© 2021 Maharaj R et al. *JAMA Network Open*.

d. eTable 1. Characteristics of ICU admissions with sepsis between 2010 and 2016

Variable	Total Sepsis	Year							
		2010	2011	2012	2013	2014	2015	2016	P value
	N=305738	N=36079	N=38643	N=41075	N=43100	N=46835	N=48685	N=51321	
Age in years									<0.001
<54	77082(25.2)	9540(26.4)	9950(25.8)	10000(24.3)	10411(24.1)	11464(24.5)	12237(25.1)	13480(26.2)	
54-66	77258(26.3)	9372(26.0)	9860(25.5)	10432(25.4)	10902(25.3)	11711(25.0)	12103(24.9)	12878(25.1)	
67-76	79820(26.1)	8967(24.9)	9952(25.8)	10562(25.7)	11284(26.1)	12455(26.6)	12920(26.4)	13680(26.7)	
>76	71578(23.4)	8200(22.7)	8881(23.0)	10081(24.5)	10503(24.3)	11205(23.9)	11425(23.5)	11283(22.0)	
Male gender	167895(54.9)	19638(54.4)	21225(54.9)	22435(54.6)	23541(54.6)	25699(55.3)	26922(55.3)	28435(55.4)	0.023
Ethnicity									<0.001
White	277787(90.9)	33153(92.0)	35534(92.1)	37721(92.0)	29429(91.5)	42474(90.7)	43718(89.8)	45758(89.2)	
Asian	10723(3.5)	1168(3.2)	1145(3.0)	1304(3.2)	1465(3.4)	1742(3.7)	1890(3.9)	2009(3.9)	
Black	6192(2.0)	663(1.8)	711(1.8)	773(1.9)	851(2.0)	1010(2.2)	1041(2.1)	1143(2.2)	
Mixed/other	10855(3.6)	1046(2.9)	1168(3.0)	1227(3.0)	1355(3.1)	1609(3.4)	2038(4.2)	2413(4.7)	
Comorbidities									
Cardiac	5375(1.8)	610(1.7)	616(1.6)	740(1.8)	708(1.7)	869(1.9)	894(1.8)	938(1.9)	0.012
Respiratory	13500(4.4)	1690(4.7)	1700(4.4)	1802(4.4)	1887(4.4)	2048(4.4)	2191(4.5)	2182(4.3)	0.092
ESRD*	5997(2.0)	669(1.9)	717(1.9)	728(1.8)	816(1.9)	925(2.0)	1049(2.2)	1093(2.1)	<0.001
Liver	7049(2.3)	796(2.2)	880(2.3)	945(2.3)	970(2.3)	1119(2.4)	1143(2.4)	1196(2.3)	0.652
Haematological malignancy	10828(3.6)	1194(3.2)	1416(4.0)	1426(3.5)	1474(3.43)	1713(3.7)	1738(3.6)	1867(3.7)	0.042
Metastatic cancer	7492(2.5)	729(2.0)	899(2.3)	935(2.3)	1035(2.4)	1226(2.6)	1281(2.6)	1387(2.7)	<0.001
Level of dependency prior to acute hospitalization									<0.001
Independent	208339(68.4)	25142(70.0)	26998(70.2)	28091(68.7)	29319(68.3)	31240(67.0)	32797(67.6)	34752(67.9)	
Some assistance	90536(29.7)	10147(28.2)	10848(28.2)	12065(29.5)	12843(29.9)	14564(31.2)	14748(30.4)	15321(30.0)	
Total dependence	5677(1.9)	620(1.7)	621(1.6)	748(1.8)	775(1.8)	839(1.8)	994(2.1)	1080(2.1)	
Usual residence prior to hospitalization									0.755
Home	296726(97.1)	35017(97.0)	37507(97.1)	39836(97.0)	41785(97.0)	45406(97.0)	47309(97.2)	49866(97.2)	
Work or non-health related institution	638(0.2)	75(0.2)	84(0.2)	90(0.2)	88(0.2)	94(0.2)	102(0.2)	105(0.2)	
Nursing home, hospice or health related institution	7327(2.4)	857(2.3)	914(2.4)	1002(2.4)	1085(2.5)	1169(2.5)	1126(2.3)	1174(2.3)	
No fixed address	1057(0.4)	134(0.4)	139(0.4)	149(0.4)	142(0.3)	166(0.4)	150(0.3)	177(0.3)	
IMD** quintile									0.075
I	77808(25.6)	9321(26.0)	9894(25.8)	10358(25.3)	11141(26.0)	12016(25.9)	12149(25.2)	12929(25.4)	
II	65377(21.5)	7608(21.2)	8357(21.8)	8833(21.6)	9146(21.3)	9922(21.3)	10540(21.8)	10971(21.6)	
III	59516(19.9)	7125(19.9)	7535(19.6)	8058(19.7)	8254(19.2)	9073(19.5)	9569(19.8)	9902(19.5)	
IV	53094(17.5)	6183(17.2)	6593(17.2)	7273(17.8)	7473(17.4)	8205(17.7)	8376(17.4)	8991(17.7)	

© 2021 Maharaj R et al. *JAMA Network Open*.

V	47809(15.8)	5663(15.8)	6025(15.7)	6360(15.9)	6831(15.9)	7255(15.6)	7620(15.8)	8055(15.8)	
APACHE II score, mean(95% CI)	18.4(18.3-18.4)	18.6(18.5-18.7)	18.6(18.5-18.7)	18.5(18.4-18.6)	18.4(18.3-18.5)	18.4(18.3-18.4)	18.2(18.2-18.3)	18.0(17.9-18.0)	<0.001
ICNARC score, mean(95% CI)	20.6(20.6-20.7)	21.4(21.2-21.4)	21.1(21.0-21.2)	20.9(20.8-21.0)	20.8(20.7-20.9)	20.5(20.4-20.5)	20.3(20.3-20.4)	20.0(19.9-20.1)	<0.001
ICNARC predicted probability of death, mean% (95% CI)	29.6(29.5-29.7)	30.9(30.6-31.2)	30.6(30.2-30.8)	30.4(30.1-30.6)	30.2(30.0-30.4)	29.4(29.1-29.7)	28.9(28.6-29.0)	27.8(27.5-28.0)	<0.001
Renal failure in the first 24 hours	25731(8.6)	3348(9.5)	3588(9.5)	3593(8.9)	3657(8.6)	3826(8.3)	3888(8.1)	3831(7.6)	<0.001
Mechanical ventilation	16600(54.3)	22140(61.3)	22688(58.7)	23091(5.2)	23534(54.6)	24440(52.2)	24644(50.6)	25469(48.6)	<0.001
Septic shock	58911(19.3)	7740(21.5)	8008(20.7)	8272(20.1)	8426(19.6)	8813(18.8)	9015(18.5)	8637(16.8)	<0.001
Non-surgical	227533(74.4)	26545(73.6)	28470(73.7)	30343(73.9	32099(74.5)	34890(74.5)	36359(74.7)	38827(75.7)	<0.001
ICU length of stay in hours, median IQR	93(43-198)	94(42-210)	92(42-204)	92(42-196)	92(42-1)95)	93(44-195)	93(43-196)	96(45-195)	<0.001
Hospital length of stay in days, median IQR	15(7-32)	26(8-34)	16(8-33)	16(7-32)	16(7-32)	15(8-31)	15(7-30)	15(7-29)	<0.001
Transferred in	12938(4.2)	1707(4.7)	1801(4.7)	1761(4.3)	1838(4.3)	1887(4.0)	1924(4.0)	2020(3.9)	<0.001
Readmission	20451(6.7)	2404(6.7)	2863(7.4)	2905(7.1)	2966(6.9)	3114(6.7)	3115(6.4)	3084(6.0)	<0.001
Unadjusted ICU mortality	62277(22.8)	8121(25.3)	8208(24.1)	8598(23.5)	8830(23.0)	9172(21.9)	9543(21.8)	9805(21.2)	<0.001
Unadjusted Hospital mortality	86728(31.9)	11326(35.6)	11413(33.7)	11945(32.9)	12325(32.2)	12831(30.7)	13331(30.6)	13557(29.5)	<0.001

*ESRD= end stage renal disease

**IMD= Index of Multiple Deprivation 2011

For categorical variables a Chi squared test was used. The null hypothesis was that there is no difference in the distribution of responses to the outcome across comparison groups. For continuous variables we use the ANOVA to analyse the differences in means between groups.

© 2021 Maharaj R et al. *JAMA Network Open*.

eTable 2 ICU characteristics between 2010 and 2016

Variable	Number								
		2010	2011	2012	2013	2014	2015	2016	p-value
ICU beds, median (IQR)	13(9-18)	11(8-16)	12(9-17)	12(9-17)	13(9-19)	13(9-19)	14(10-12)	15(10-20)	<0.001
Occupancy %, median (IQR)	73.5(67.9-79.5)	71.0(65.1-76-2)	71.6(64.7-77.7)	72.6(67.3-79.1)	74.2(68.7-80.1)	73.4(68.2-79.7)	75.0(69.8-80.0)	75.8(80.5-80.8)	<0.001
Sepsis volume, median (IQR)	242(177-334)	213(156-274)	221(160-295)	233(170-306)	253(175-338)	258(186-351)	259(196-350)	276(200-382)	<0.001
Non-sepsis volume, median (IQR)	497(346-747)	432(295-607)	472(325-684)	493(346-722)	496(340-774)	519(368-819)	527(377-871)	555(382-819)	<0.001
Total volume, median (IQR)	742(533-1087)	666(459-911)	691(492-994)	729(519-1085)	732(519-1175)	774(577-1229)	785(596-1254)	827(598-1288)	<0.001

For categorical variables a Chi squared test was used. The null hypothesis was that there is no difference in the distribution of responses to the outcome across comparison groups. For continuous variables we use the ANOVA to analyse the differences in means between groups.

© 2021 Maharaj R et al. *JAMA Network Open*.

4. Additional results

a. eFigure 3. Odds ratio for mortality with volume as quartiles.

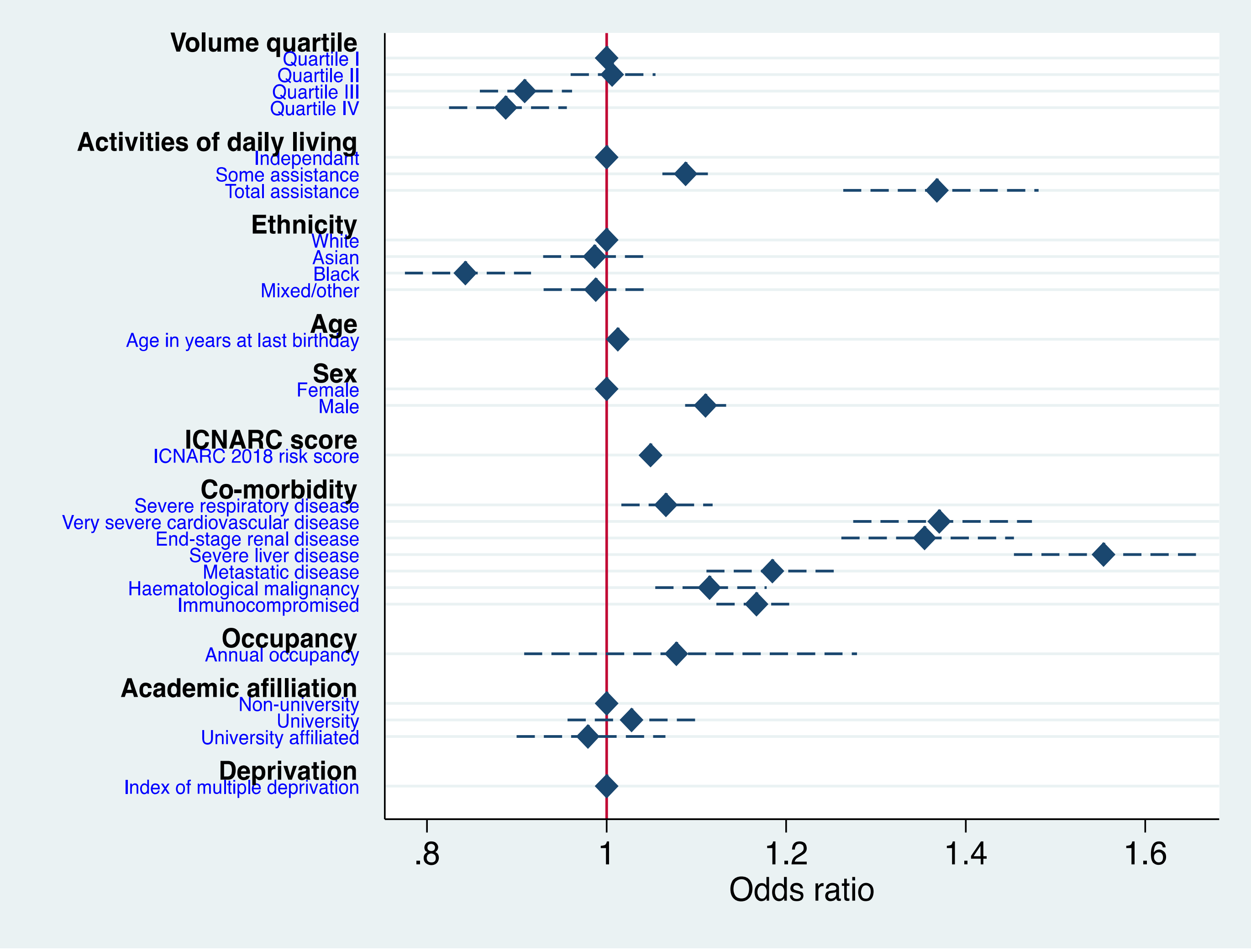

© 2021 Maharaj R et al. *JAMA Network Open*.

b. eTable 3. Testing the statistical significance between models of increasing complexity with volume defined as a restricted cubic spline.

**Sensitivity Analysis**	Chi2	df	p-value
Sepsis expressed cubic splines			
3 knots	16.67	2	0.0002
4 knots	18.65	3	0.0003
5 knots	18.71	4	0.0009
6 knots	20.83	5	0.0009

© 2021 Maharaj R et al. *JAMA Network Open*.

c._eFigure 4. The within-ICU variation across between 2010 and 2016.

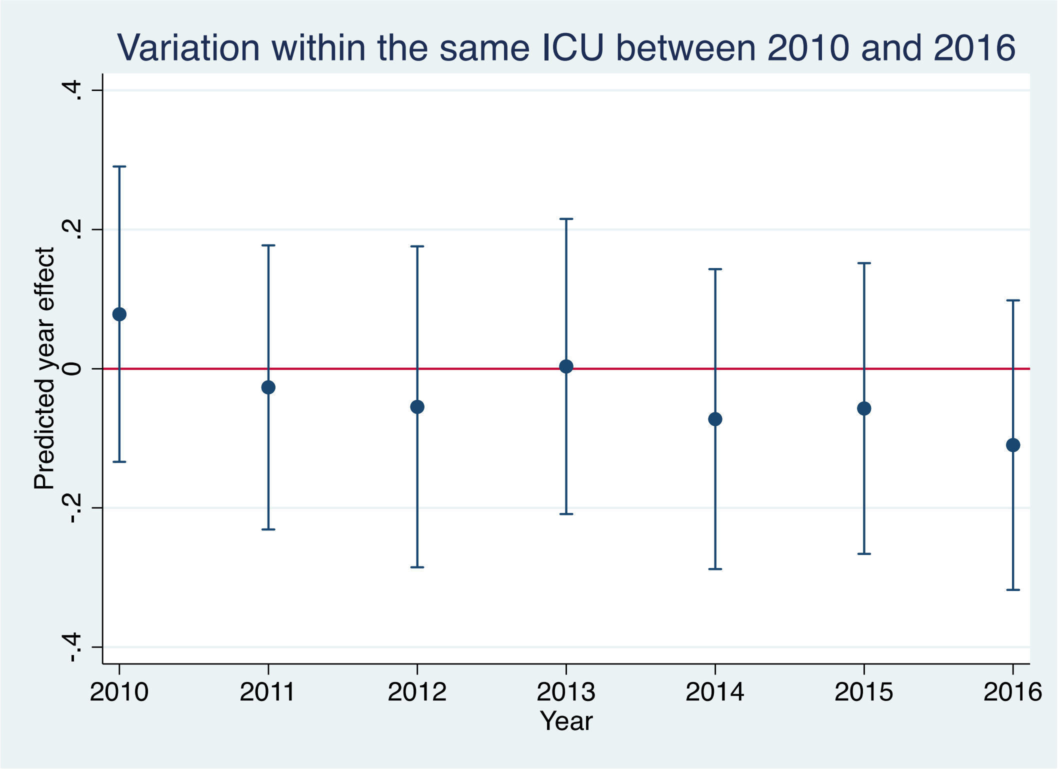

The figure represents variation in mortality not explained by measurable characteristics within the same ICU over the study period.

© 2021 Maharaj R et al. *JAMA Network Open*.

d. eFigure 5. Subgroup analysis: (A) Mechanical ventilation (B) ICNARC predicted mortality >30% (C) Renal replacement therapy within 24 hours of admission (D) Septic shock (E) Non-surgical patients (F) Readmissions included

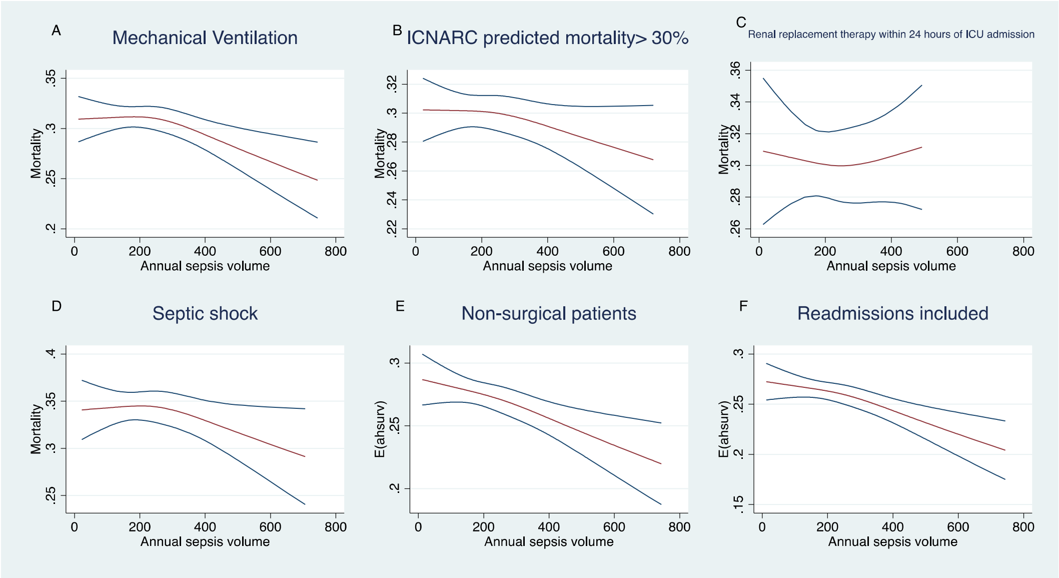

There was no enhanced reduction in mortality from ICU volume in patients with more severe illness as characterised by the subgroup of patients with mechanical ventilation, ICNARC predicted mortality >30%, renal replacement therapy within 24 ours of admission and septic shock. The reduction of mortality was consistent in the subgroup of patients with sepsis not requiring surgical procedures. The inclusion of the outcome from subsequent ICU readmission episodes did not impact the observed volume-outcome relationship.

© 2021 Maharaj R et al. *JAMA Network Open*.

5. Sensitivity analysis

a. Fractional polynomial and selection procedure

i. eTable 4. Output from model fitting procedure for fractional polynomial showing deviance and powers of model

Model	Sepsis volume	df	Deviance	Deviance difference	P-value	Powers
Model 1	Omitted	0	232492.13	16968	<0.0001	
	Linear	1	232475.76	0.596	0.440	1
	M=1	2	232475.16	0.000	-	2
Model 2	Omitted	0	232492.13	17.735	<0.001	
	Linear	1	232475.76	2.531	0.282	1
	M=1	2	232475.16	1.934	0.164	2
	M=2	3	232473.23	0.00	-	3 3
Model 3	Omitted	0	232492.13	233.253	0.001	
	Linear	1	232475.76	6.899	0.230	1
	M=1	2	232475.16	6.069	0.179	2
	M=2	3	232473.23	4.369	0.226	3 3
	M=3	6	232468.88	0.00	-	-2 0 0

The general formulation of fractional polynomials is:

$$x^{(p_1,p_2,\ldots,p_m)\prime}\beta = \beta_0 + \beta_1 x^{(p_1)} + \beta_2 x^{(p_2)} + \cdots + \beta_m x^{(p_m)}$$

x^0 is interpreted as $\ln(x)$ and repeat powers are multiplied by $\ln(x)$, where $x$indicates ICU volume.

© 2021 Maharaj R et al. *JAMA Network Open*.

The models are constructed iteratively. In Model 1, the highest power is 2 thus the fractional polynomial with the dimension $M = 1$ would be $\beta_1 x^2$. The linear model included as part of the selection procedure would be $\beta_1 x$. In Model 2 the highest FP where the dimension $M = 2$ would be $\beta_1 x^3 + \beta_2 x^3 \ln(x)$ and included the preceding iterations of Model 1 for comparison. The highest power in Model 3 where the dimension $M = 3$ is $\beta_1 x^{-2} + \beta_2 \ln(x) + \beta_3 \ln(x) \ln(x)$.

The selection procedure compares the highest FP model with the model that omits the x variable If significant, the selection procedure, then compares the FP model with the linear model. If this in turn is significant, the selection procedure then compares the most complex FP model with the next model down the list of iterations. A more complex model is chosen if that model fits the data better based on significance criteria.

© 2021 Maharaj R et al. *JAMA Network Open*.

ii. eFigure 6. Functional form of fractional polynomial models showing Model 1, Model 2 and Model 3.

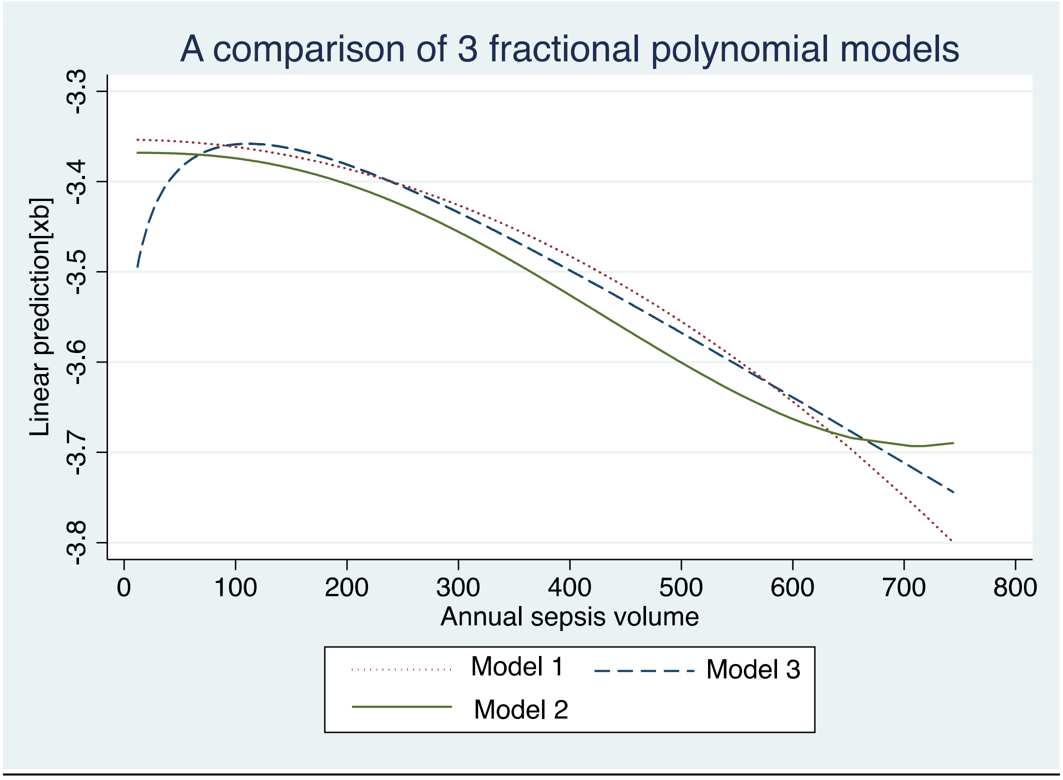

The graph describes the functional form of the FP models where $Model\,1 = \beta_1 x^2$, $Model\,2 = \beta_1 x^3 + \beta_2 x^3 \ln{(x)}$ and $Model\,3 = \beta_1 x^{-2} + \beta_2 \ln{(x)} + \beta_3 \ln(x) \ln{(x)}$. The models are in increasing complexity. The models of higher complexity show less stable results at the extremes of ICU volume [13].

© 2021 Maharaj R et al. *JAMA Network Open*.

iii. eTable 5. A comparison of the log likelihood and information criteria for the linear, fractional polynomial and restricted cubic spline models.

**Model**	**df**	**Log likelihood**	**AIC**	**BIC**
Categorical	25	-116234	232518.9	232780.4
Linear	23	-116237.9	232521.8	232762.4
Restricted cubic splines				
3 knots	24	-116237.1	232522.2	232773.3
4 knots	25	-116236.1	232522.1	232783.7
5 knots	26	-116236.0	232524.1	232796.1
6 knots	27	-116234.9	232523.9	232806.3
Fractional polynomial				
Model 1	23	-116237.6	232521.2	232761.8
Model 2	23	-116236.6	232519.2	232843.3
Model 3	25	-116234.4	232518.9	232780.4

The model that minimises both AIC and BIC is preferred but no model does this for this data. At higher sample sizes the AIC may select the model that is too complex and the BIC has a higher probability of selecting the true model[14]. As the sample size increases, the BIC offers more general consistency. Using likelihood alone as a model selection criterion in not advised as it will tend to select overly-paramaterized models[15]. The 3-knot model has the lowest BIC is the considered best fitting restricted cubic spline. In terms of FP models, Model 1 minimises the BIC. There are several competing models, one of which can be categorially declared as better than the rest.

© 2021 Maharaj R et al. *JAMA Network Open*.

b. E-values

ii. Rationale

Regression analyses provide some control for measured confounding but there is potential for unmeasured confounding. Being unmeasured, it is a challenge to assess what impact this latter form confounding might have on the analyses[16]. Ding and VanderWeele have proposed a bounding factor approach to assess the sensitivity of the results to unmeasured confounders[16]. They define a bounding factor (E-value) which is the minimum strength of association on a risk-ratio scale that the unmeasured confounder would need to have a material effect on the results[17]. The E-value describes two parameters, the risk ratio for the confounder-outcome relationship and the risk ratio of the exposure confounder relationship. There is no absolute threshold for an E-value and the reader must then assess whether the magnitude of the unmeasured confounder is plausible[17]. In this study, the OR for acute hospital mortality for quartile IV compared with quartile I of sepsis volume was 0.89 with a 95% CI of 0.82-0.96.The E-value for this point estimate is 1.31 and for the lower confidence limit is 1.17. This would mean that the observed OR could be explained by the presence of unmeasured confounding associated with both the exposure and the outcome by a risk ratio of 1.31, above and beyond measured confounders. Although a RR of 1.31 appears modest, in the current study this seems unlikely because this would imply that the unmeasured

© 2021 Maharaj R et al. *JAMA Network Open*.

covariates would have to be similar in magnitude to measured covariates like severe respiratory disease or haematologic malignancy. The Case Mix Program is a high-quality clinical database, and we suggest that an unmeasured confounder of this magnitude is unlikely.

© 2021 Maharaj R et al. *JAMA Network Open*.

ii. eFigure 7. Value of the joint minimum strength of association that an unmeasured confounder must have with both an increase in ICU sepsis volume and acute hospital mortality to explain away the volume outcome relationship, expressed as a risk ratio

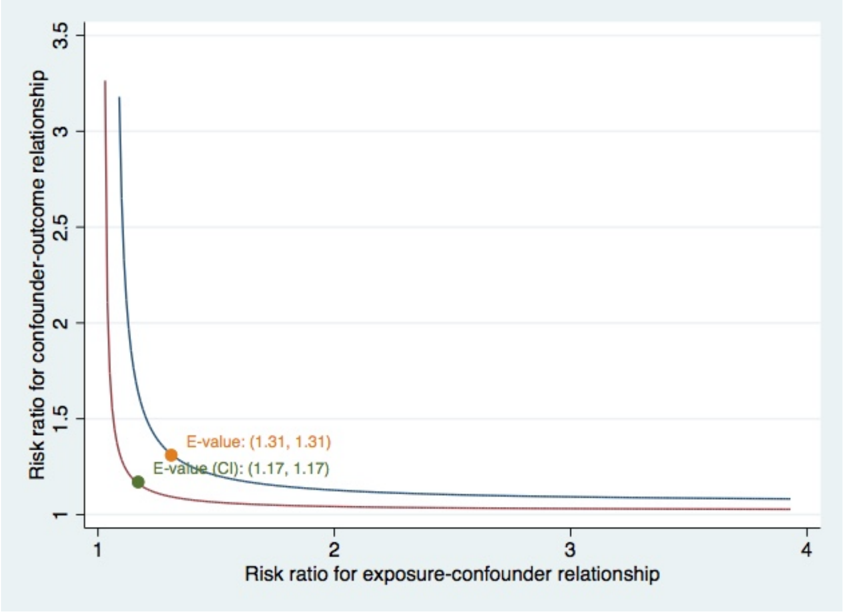

The E-value describes 2 parameters, the risk ratio for the confounder-outcome relationship and the risk ratio of the exposure confounder relationship. The area above the two lines are the joint exposure confounder risk ratio and confounder outcome risk ratio that would be required to explain the observed treatment effect.

© 2021 Maharaj R et al. *JAMA Network Open*.

c. Checking for exogeneity ICU volume

i. Rationale

One of the assumptions of the multilevel random effects model is exogeneity- specifically in our model it would require that ICU volume is uncorrelated with the ICU-level random effect. [18]. The random effects model assumes that the within- and between ICU effects are the same and uses a weighted average of the within and between ICU effects in estimation. The p-value tests the significance of this difference. This is a regression-based alternative to the Hausman test for endogeneity of a regressor[19, 20].

Stated simply, the exogeneity assumption holds i.e., the random effects model of no correlation between the random effect and the level-1 covariates holds[20]. We describe these results as part of a robustness check to our model assumptions.

ii. eTable 6. Within and between cluster effects of ICU volume test for exogeneity.

**Variable**	**Coefficient**	**Standard Error**	**p-value**
Sepsis Volume			
Quartile I (W)	0.1474	0.0397	
Quartile II (W)	0.1541	0.3254	
Quartile III(W)	0.0371	0.0276	
Quartile IV(W)	*(omitted)*		
Quartile I (B)	0.0442	0.0607	
Quartile II (B)	0.0207	0.0634	
Quartile III(B)	0.0146	0.0678	
Quartile IV(B)	*(omitted)*		
**Quartile I (B-W)**	**0.0441**	**0.0607**	**0.467**
**Quartile II (B-W)**	**0.0207**	**0.0634**	**0.745**
**Quartile III(B-W)**	**0.0146**	**0.0678**	**0.830**
Quartile IV(B-W)	*(omitted)*		
Restricted Cubic Spline			
Spline-1 (W)	-0.0003	0.00003	
Spline-2(W)	-0.0006	0.00003	
Spline-1 (B)	-0.0003	0.0004	
Spline-2 (B)	0.0003	0.0005	
**Spline-1 (B-W)**	$\mathbf{4.23}e^{-6}$	**0.0004**	**0.9930**
**Spline-2(B-W)**	**0.0009**	**0.0006**	**0.1551**

© 2021 Maharaj R et al. *JAMA Network Open*.

(W)= within cluster; (B)= between cluster; (B-W) = difference in between and within-cluster effects.

The lack of statistical significance, as highlighted by the highlighted p values, in the between- and within-cluster effects for ICU volume imply a lack of correlation in the ICU volume and the ICU random effect, in support of the assumption that ICU volume is exogenous.

© 2021 Maharaj R et al. *JAMA Network Open*.

References

1. Goldstein H: **Hierarchical Data Modeling in the Social Sciences**. *Journal of Educational and Behavioral Statistics* 1995, **20**(2):201-204.
2. Goldstein H, Spiegelhalter DJ: **League Tables and Their Limitations: Statistical Issues in Comparisons of Institutional Performance**. *Journal of the Royal Statistical Society Series A (Statistics in Society)* 1996, **159**(3):385-443.
3. Orsini N, Greenland S: **A procedure to tabulate and plot results after flexible modeling of a quantitative covariate**. *Stata Journal* 2011, **11**(1):1-29.
4. Lusa L, Ahlin C: **Restricted cubic splines for modelling periodic data**. *PLoS One* 2020, **15**(10):e0241364.
5. Smith OM, Chant C, Burns KEA, Kaur M, Ashraf S, DosSantos CC, Hwang SW, Friedrich JO: **Characteristics, clinical course, and outcomes of homeless and non-homeless patients admitted to ICU: A retrospective cohort study**. *PLoS One* 2017, **12**(6):e0179207.
6. Ginde AA, Moss M, Shapiro NI, Schwartz RS: **Impact of older age and nursing home residence on clinical outcomes of US emergency department visits for severe sepsis**. *J Crit Care* 2013, **28**(5):606-611.
7. **English indices of deprivation** [https://www.gov.uk/government/collections/english-indices-of-deprivation]
8. Knaus WA, Draper EA, Wagner DP, Zimmerman JE: **APACHE II: a severity of disease classification system**. *Crit Care Med* 1985, **13**(10):818-829.
9. Harrison DA, Rowan KM: **Outcome prediction in critical care: the ICNARC model**. *Curr Opin Crit Care* 2008, **14**(5):506-512.
10. Harrison DA, Parry GJ, Carpenter JR, Short A, Rowan K: **A new risk prediction model for critical care: the Intensive Care National Audit & Research Centre (ICNARC) model**. *Crit Care Med* 2007, **35**(4):1091-1098.
11. Ferrando-Vivas P, Jones A, Rowan KM, Harrison DA: **Development and validation of the new ICNARC model for prediction of acute hospital mortality in adult critical care**. *J Crit Care* 2017, **38**:335-339.
12. Levy MM, Artigas A, Phillips GS, Rhodes A, Beale R, Osborn T, Vincent JL, Townsend S, Lemeshow S, Dellinger RP: **Outcomes of the Surviving Sepsis Campaign in intensive care units in the USA and Europe: a prospective cohort study**. *Lancet Infect Dis* 2012, **12**(12):919-924.
13. Royston P: **Model selection for univariable fractional polynomials**. *Stata J* 2017, **17**(3):619-629.
14. Hastie T, Friedman J, Tisbshirani R: **The Elements of statistical learning : data mining, inference, and prediction**. New York: Springer; 2018.
15. Gelfand AE, Dey DK: **Bayesian Model Choice: Asymptotics and Exact Calculations**. *Journal of the Royal Statistical Society Series B (Methodological)* 1994, **56**(3):501-514.
16. Ding P, VanderWeele TJ: **Sensitivity Analysis Without Assumptions**. *Epidemiology* 2016, **27**(3):368-377.
17. VanderWeele TJ, Ding P: **Sensitivity Analysis in Observational Research: Introducing the E-Value**. *Ann Intern Med* 2017, **167**(4):268-274.

© 2021 Maharaj R et al. *JAMA Network Open*.

18. Schunck R, Perales F: **Within- and Between-cluster Effects in Generalized Linear Mixed Models: A Discussion of Approaches and the Xthybrid command**. *The Stata Journal* 2017, **17**(1):89-115.
19. Cameron AC, Triverdi PK: **Microeconometrics using Stata**. College Station, Tex: Stata Press; 2010.
20. Allison PD: **Fixed Effects Regression Models**. Thousand Oaks, Calif.: Sage Publications; 2009.
